# Cardiac and renal function interactions in heart failure with reduced ejection fraction: A mathematical modeling analysis

**DOI:** 10.1371/journal.pcbi.1008074

**Published:** 2020-08-17

**Authors:** Hongtao Yu, Sanchita Basu, K. Melissa Hallow

**Affiliations:** 1 School of Chemical, Materials, and Biomedical Engineering, University of Georgia, Athens, Georgia, United States of America; 2 Department of Epidemiology and Biostatistics, University of Georgia, Athens, Georgia, United States of America; University of Michigan, UNITED STATES

## Abstract

Congestive heart failure is characterized by suppressed cardiac output and arterial filling pressure, leading to renal retention of salt and water, contributing to further volume overload. Mathematical modeling provides a means to investigate the integrated function and dysfunction of heart and kidney in heart failure. This study updates our previously reported integrated model of cardiac and renal functions to account for the fluid exchange between the blood and interstitium across the capillary membrane, allowing the simulation of edema. A state of heart failure with reduced ejection fraction (HF-rEF) was then produced by altering cardiac parameters reflecting cardiac injury and cardiovascular disease, including heart contractility, myocyte hypertrophy, arterial stiffness, and systemic resistance. After matching baseline characteristics of the SOLVD clinical study, parameters governing rates of cardiac remodeling were calibrated to describe the progression of cardiac hemodynamic variables observed over one year in the placebo arm of the SOLVD clinical study. The model was then validated by reproducing improvements in cardiac function in the enalapril arm of SOLVD. The model was then applied to prospectively predict the response to the sodium-glucose co-transporter 2 (SGLT2) inhibitor dapagliflozin, which has been shown to reduce heart failure events in HF-rEF patients in the recent DAPAHF clinical trial by incompletely understood mechanisms. The simulations predict that dapagliflozin slows cardiac remodeling by reducing preload on the heart, and relieves congestion by clearing interstitial fluid without excessively reducing blood volume. This provides a quantitative mechanistic explanation for the observed benefits of SGLT2i in HF-rEF. The model also provides a tool for further investigation of heart failure drug therapies.

## Author summary

Recently, the DAPAHF and EMPA-REG clinical trials showed that the Sodium-Glucose Cotransporter-2 Inhibitor (SGLT2i) class of drugs are beneficial in reducing the heart failure with reduced ejection fraction (HFrEF) hospitalization and lowering the risk of worsening HF or death. Despite the beneficial outcomes observed clinically, the hemodynamic mechanisms of SGLT2i underlying the DAHAHF results remain unclear. Mathematical modeling has long been used to investigate the integrated organ function and dysfunction and understand the effects of interventions. A proposed cardio-renal mathematical model was adopted and improved to capture the state of HFrEF and cardiac therapeutic responses. Validation studies were carried out by reproducing improvements in cardiac functions in the enalapril arm of the SOLVD clinical trial. The influences of SGLT2i on cardiac and renal functional interactions were investigated. It was observed that SGLT2i reduces left ventricle (LV) end-diastolic volume/pressure (EDV/EDP), and that this preload reduction slows cardiac remodeling and progression over one year. It further presents that SGLT2i may reduce interstitial congestion without inducing large reductions in blood volume.

This is a *PLOS Computational Biology* Methods paper.

## Introduction

Chronic heart failure (HF) is a condition in which the heart is incapable of preserving a sufficient cardiac output (CO) to reach the metabolic requirements of peripheral organs [1]. HF with reduced ejection fraction (HF-rEF) is characterized by a left ventricle ejection fraction (LVEF) less than 40%, suppressed CO, elevated cardiac filling pressure, and progressive eccentric remodeling of the heart. HF-rEF is often a consequence of ischemic damage to the cardiac muscle, although HF-rEF may also result from valvular disease, hypertension, or idiopathic causes [2–6]. Inadequate organ perfusion due to depressed CO activates neurohormonal mechanisms, such as the renin-angiotensin-aldosterone-system (RAAS). However, excessive volume retention increases both preload and afterload on the heart, causing detrimental cardiac remodeling. Excessive preload also leads to elevated venous pressure and the development of peripheral edema and pulmonary congestion [7,8]. These factors contribute to the progressive worsening of HF over time, usually with repeated and costly hospitalizations [9].

The complexity of integrated functions of the heart, kidney, and neurohormonal mechanisms makes it challenging to understand or predict the interplay between the heart and kidney, especially when one or both are in a state of dysfunction. In particular, many therapies that treat HF act through renal mechanisms, and the effects of these therapies on cardiac functions can be unexpected or unintuitive. For instance, recent studies have shown that sodium-glucose co-transporter 2 inhibitors (SGLT2i), which act primarily by inhibiting glucose reabsorption in the proximal tubule of the kidney, significantly reduce HF hospitalization and cardiovascular death in patients with HF-rEF [10]. Studies have also shown that SGLT2i reduces HF events in diabetics with increased cardiovascular risk [11,12]. However, the mechanism(s) by which SGLT2i induces these cardiac effects remain incompletely understood. In particular, the effects of SGLT2i on cardiac hemodynamics in HF have not been reported. Difficulties in obtaining comprehensive clinical measures of cardiac and renal hemodynamics in fragile HF patients add to this challenge.

Mathematical modeling is one means to investigate integrated organ function and dysfunction and to understand the effects of interventions. Lumped parameter models have frequently been utilized to model the hemodynamic characteristics of the heart [13–19], computing the pressure and volume of heart chambers through a time-dependent elastance by prescribing two constants of active and passive elastance of heart chambers [13–16]. However, these models assume constant blood volume and thus ignore the role of the kidney. They also do not allow simulation of changes in cardiac structure in response to changes in loading. Detailed computational models that separately describe cardiac [20,21] and renal [22–27] functions have been developed, but these models also do not consider the interdependency of the heart and kidney. The “whole-body” mathematical model of blood volume regulation established by Guyton et al. incorporated circulatory dynamics, neurohormonal controls, blood fluid controls, and renal function, and demonstrated the physiological compensation that occurs when one part of the entire system is functioning abnormally [28]. Multiple updates, revisions, and improvements have been made to this model over the decades [29–34], and have been applied to investigate effects of therapies such as RAAS blockers (e.g. angiotensin-converting-enzyme inhibitor (ACEi)) and SGLT2i on blood pressure, renal hemodynamics, and body fluid regulation [29,35], and the mechanism of pressure natriuresis [36,37]. Nevertheless, the simplified representation of cardiac physiology utilized by these mathematical models [31,32,35] has limited the ability to investigate the impact of impaired cardiac function on renal function and body fluid regulation, or the impact of renally or neurohormonally-driven changes in cardiac preload and afterload on cardiac function and remodeling.

We recently developed an integrated cardiorenal model that couples previous models of renal and neurohormonal function with a model of ventricular wall mechanics [17,19]. This model also simulates cardiac remodeling using the framework demonstrated by Grossman et al, in which the cardiac muscle responds to changes in loading patterns in two distinct ways [38]. Elevated LV peak systolic stress drives increases in myocyte diameter and thickening of the vessel wall, while elevated LV end-diastolic stress drives increases in myocyte length and dilatation of the vessel wall. In pathologic states, the relative changes in these two stresses determine whether the heart walls thicken or dilate, or some combination. We have previously shown that, using this framework, the model is able to describe different patterns of hypertrophy characteristic of pressure and volume overload due to aortic stenosis and mitral regurgitation, respectively [39,40]. It was showed previously that it is able to reproduce time course and magnitude of regression of LV hypertrophy response to the natriuretic effects of losartan or atenolol therapy observed in the LIFE study in [41]. However, this previous work did not consider the states of HF.

The aims of this study were two-fold. First, we aimed to use the integrated cardiorenal model to represent states of HF-rEF, the progression of cardiac remodeling in HF-rEF over time, and improvements with ACEi therapy. We then aimed to prospectively simulate the effect of SGLT2i on cardiac hemodynamics and volume status, in order to better understand the mechanisms underlying observed reductions in HF hospitalization and cardiovascular death observed with SGLT2i.

## Results

### Baseline model

As shown in Table 1, simulated steady-state values for key clinically measurable variables fall within the normal range for healthy subjects.

**Table 1:** Comparison of simulated hemodynamic variables at baseline with clinical values in healthy and HF subjects.

Variables	Units	Healthy		HF-rEF	
		Simulated	Normal Range	Simulated	Clinically Observed
Cardiac Output	L/min	4.97	4–8	4.92	5±1.2[42]; 4[43]; 4.4[44]; 4.65±1.2[45];
Heart Rate	Beats/min	70	60–100	75	72[46]; 79±14[43]; 69[44];
Ejection fraction	%	71	55–75	18	26.1±12.27%[46]; 31[47]; 33± 5[44]; 21 ± 6[43];
Stroke volume	mL	71	50–100	65.27	66±16[42]; 51[43]; 69[44];
Left ventricle end diastolic volume	mL	95	62–120	358.67	192[47]; 243[44]; 259±98[43]; 368[42];
Left ventricle end systolic volume	mL	25	27–43	293.40	137[47]; 168[44]; 208±90[43]; 280[42];
Left ventricle end diastolic pressure	mmHg	6.5	5–12	19.67	25±9[42];
Blood volume	L	4.9	5	5.53	6.7[45]; 5.528[48];
Interstitial fluid volume	L	15.0	15	23.47	17.4[45]; 16.4±3.8[49];
Glomerular filtration rate	ml/min	95	>90	88.66	91.3±23 [50]; 74±20 [10]
Mean arterial pressure	mmHg	84.4	80–90	96.04	91±10[46]; 90±9[44]; 94±12[42];
Systolic blood pressure	mmHg	111.78	95–130	120.95	124±10[46]; 121±10[44]; 127±17[42];
Diastolic blood pressure	mmHg	70.77	60–90	83.58	78± 10[42]; 75±10[46]; 75±8[44];

### Simulating HF-rEF

HF-rEF is associated with reduced cardiac contractility along with cardiac dilatation, usually subsequent to myocardial infarction or other ischemic injuries. In addition, patients with HF-rEF often have a history of hypertension [8]. Renal dysfunction is also a common comorbidity with HF-rEF. Thus, to simulate HF-rEF, we altered model parameters to induce mild hypertensive and renal injury, as described previously [18,30]. Table 2 summarizes the parameters altered to induce different disease states and the corresponding pathophysiologic mechanism that each parameter represents. After running to a new equilibrium, mean arterial pressure (MAP) was increased from 85 to 96.05 mmHg and glomerular filtration rate (GFR) was decreased from 96.52 ml/min to 80.82 ml/min.

**Table 2:** Parameters Altered to Produce HF-rEF, diabetic, and diabetic HF-rEF Virtual Patients.

Parameters	Description	Normal Value	Disease State Value	Units	Refer to Equations	Pathologic mechanisms
Simulation of HF-rEF						
_*C*_	Intrinsic cardiac contractility	1	0.795	–	-	Cardiac ischemic injury
C_f_	Left ventricle stiffness	11	12.2	–	Eq. S66	Ventricular stiffness due to fibrosis
R_arterial_	Arterial resistance	5e+006	6e+006	mmHg min^-1^ ml^-1^	–	Endothelial dysfunction and vascular stiffness
R_peripheral_	Peripheral resistance	1.3E+08	1.6E+08	mmHg min^-1^ ml^-1^	–	
C_venous_	Venous compliance	2E-07	1.4E-07	ml/mmHg	–	
Simulation of hypertension						
R_preaff_	Renal preafferent arteriole resistance	14	25.5	mmHg min^-1^ ml^-1^	Eq. S1	Renal vascular disease
D_aa_	Renal afferent arteriole diameter	1.65E-05	1.55E-05	m	Eq. S2	
C_DED-Kf_	Decrease in glomerular permeability	100%	80%	%	Eq. S6	Glomerulosclerosis
C_DE-N_	Decrease in # of nephrons	100%	60%	%	Eq. S5	Nephron loss
Simulation of Diabetes						
C_glu_	Average plasma glucose concentration	5.5	8	mmol/L	Eq. S7	Diabetes

**Fig 1:**
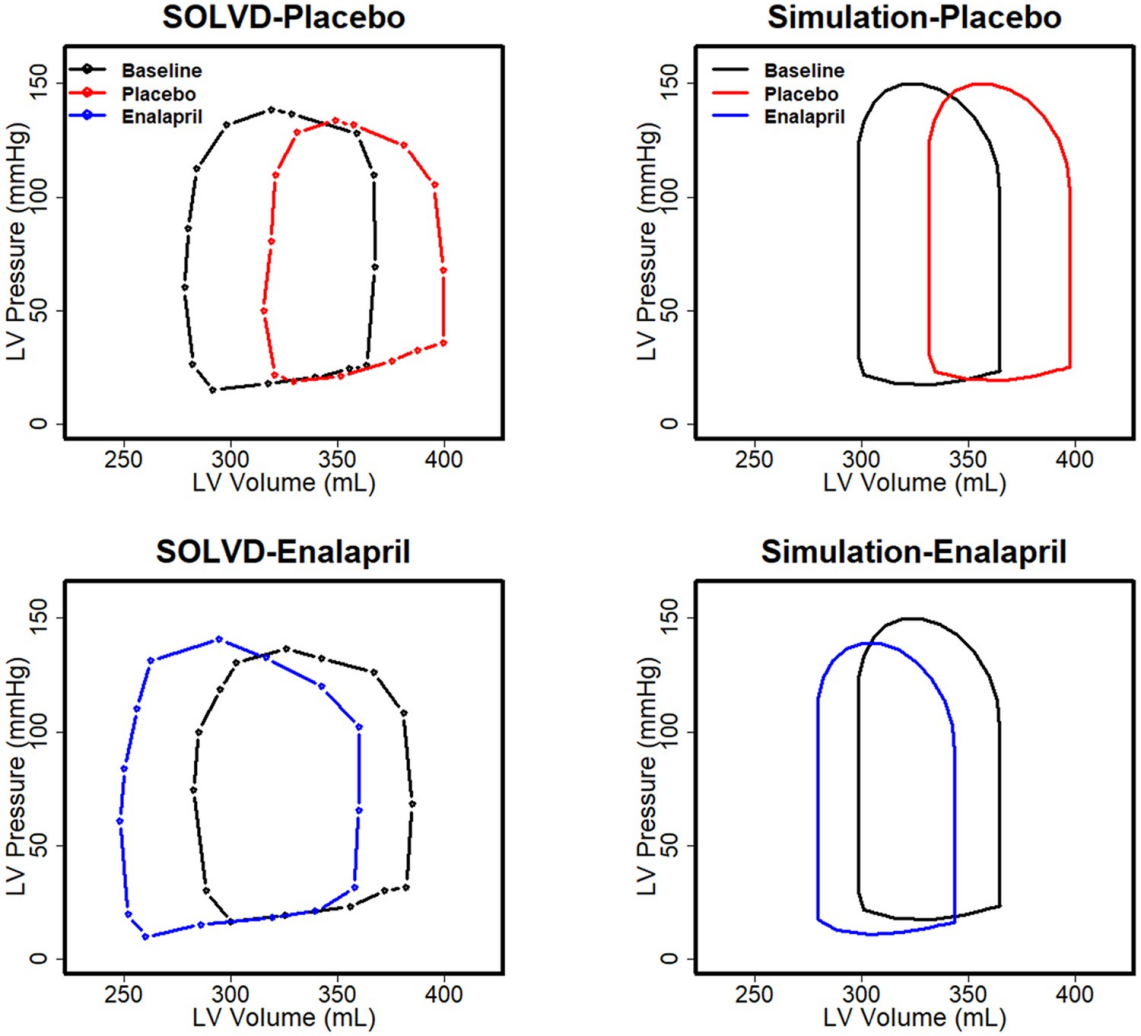
Observed (left) and simulated (right) pressure-volume loops in HF-rEF patients in the SOLVD study at baseline and after 1 year of treatment with placebo or enalapril [42]. The model reproduces the baseline P-V loop, rightward shift over time in the placebo group, and the leftward shift in the enalapril group.

Starting from this new baseline, additional parameters were altered to simulate mechanisms of HF-rEF (Table 2). These include 1) decreased cardiac contractility, representing cardiac ischemic injury; 2) increased myocyte length and diameter, representing myocyte hypertrophy and eccentric remodeling of the heart; 3) increased left ventricle (LV) stiffness due to increased myocardial fibrosis following ischemic injury; and 4) alterations in venous and arterial stiffness and resistance [51–53]. The values for the pathological parameters summarized in Table 2 were adjusted by trial and error method. Before adjusting the values, sensitivity analysis of each parameter was conducted to understand how they affect the system behavior. Subsequently, parameters were modified within their pathological ranges accordingly. The final parameter values were determined to produce behavior consistent with clinically observed HF-rEF measurements (Table 1) and to produce a pressure-volume loop (P-V loop) similar to the baseline recordings in the SOLVD study (Fig 1, black curves) [42].

### Simulating Virtual Diabetic and Diabetic HF-rEF Patients

Diabetes was defined in the model by increasing the average blood glucose concentration from the normal baseline value of 5.5 mmol/l to 8 mmol/l (Table 2). We did not consider diurnal variations in glucose concentration. Also, other non-renal cardiovascular aspects of diabetes were not considered. Two diabetic virtual patients were generated–one with normal cardiac function (all other model parameters at baseline) and one with HF-rEF (elevated glucose in addition to parameters of HF-rEF described in Table 2). In both cases, after increasing blood glucose, baseline cardiovascular variables given in Table 1 were not substantially changed.

### Simulation of the SOLVD clinical trial: Calibration and validation of HF-rEF progression and therapeutic regression

After generating the HF-rEF virtual patient, parameters defining the rate of progression of cardiac remodeling (see modeling cardiac remodeling in Methods section) were calibrated by fitting the rightward progression of the HF-rEF P-V loop over 1 year to the progression observed in the placebo arm of the SOLVD clinical trial (see trial summary in the Methods section) [42]. As shown in Fig 1 (black vs. red curves), where results show the last heartbeat for the 365^th^ day, the calibrated model reproduced 1) the left ventricle P-V loop of the SOLVD study at baseline and 2) the rightward shift in the P-V loop over one year with placebo. As validation, the response of the HF-rEF virtual patient to 1 year of treatment with the ACEi enalapril was simulated, and the resulting changes in the P-V loop were compared to those observed in the SOLVD enalapril arm. The model is capable of reproducing the leftward shift in the P-V loop with 1 year of enalapril treatment (Fig 1 blue vs. black curve). It should be noted that the shift of the PV loop towards the right observed in the placebo arm indicates the progression of HF-rEF. The designation of the pathological parameters shown in Table 2 produces a state of HF-rEF in which the heart remodels continuously. The baseline (black curve in Fig 1) was established by running the simulation to a point at which the P-V loop is similar to the baseline P-V loop in SOLVD. The placebo and ACEi cases were then simulated forward from this point for 1 year, without and with treatment.

**Fig 2:**
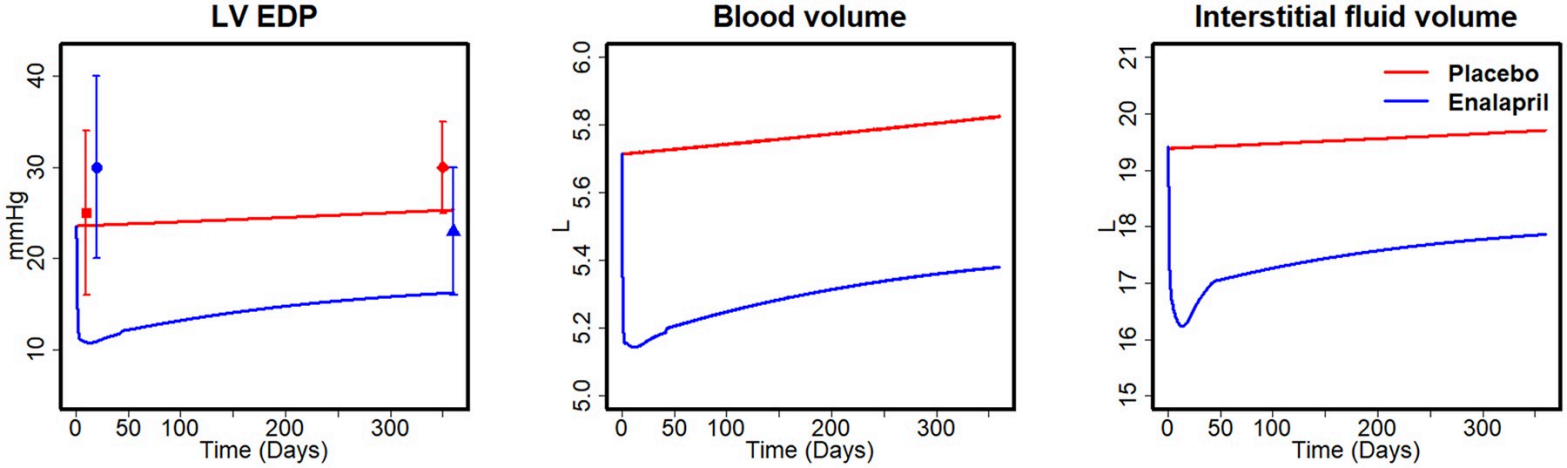
Simulated changes in LV EDP, BV, and IFV between placebo and enalapril in SOLVD. Data points for mean LV EDP from SOLVD study [42]: Square (■) baseline of placebo; diamond (♦): placebo after 1 year; circle (●): baseline of enalapril; triangle (▲): enalapril after 1 year.

As shown in Fig 2, the baseline LV end-diastolic pressure (EDP) in the placebo arm is in a good agreement with the measurement data in SOLVD. Although the baseline LVEDP was lower than that observed in the ACEi arm in SOLVD, the predicted change in LVEDP with ACEi agrees with the observed changes (Simulation: from 23.6 to 16.3 mmHg *vs*. SOLVD: from 30 to 25 mmHg over 1 year). In the model, this reduction was driven by the natriuretic effect of ACEi, which reduced blood volume (BV) and interstitial fluid volume (IFV) by 10.0% and 16.2%, respectively, within the first month. Over the following months, BV and IFV began to rise again, but the difference between the treatment and placebo group was maintained, which is consistent with the progressive trends observed in SOLVD [42].

### Prospective simulation of HF-rEF response to SGLT2 inhibition

We have previously modeled the mechanisms of action of the SGLT2i dapagliflozin in an earlier version of the model that utilized a simpler representation of the cardiac function[35]. To ensure that the integrated cardiorenal model still accurately describes the response to SGLT2i, we confirmed the model still reproduces clinical measures of dapagliflozin response, including changes in urinary glucose, sodium, and water excretion, as well as serum creatinine plasma sodium concentrations over 7 days (refer to supplemental S3 Fig).

**Fig 3:**
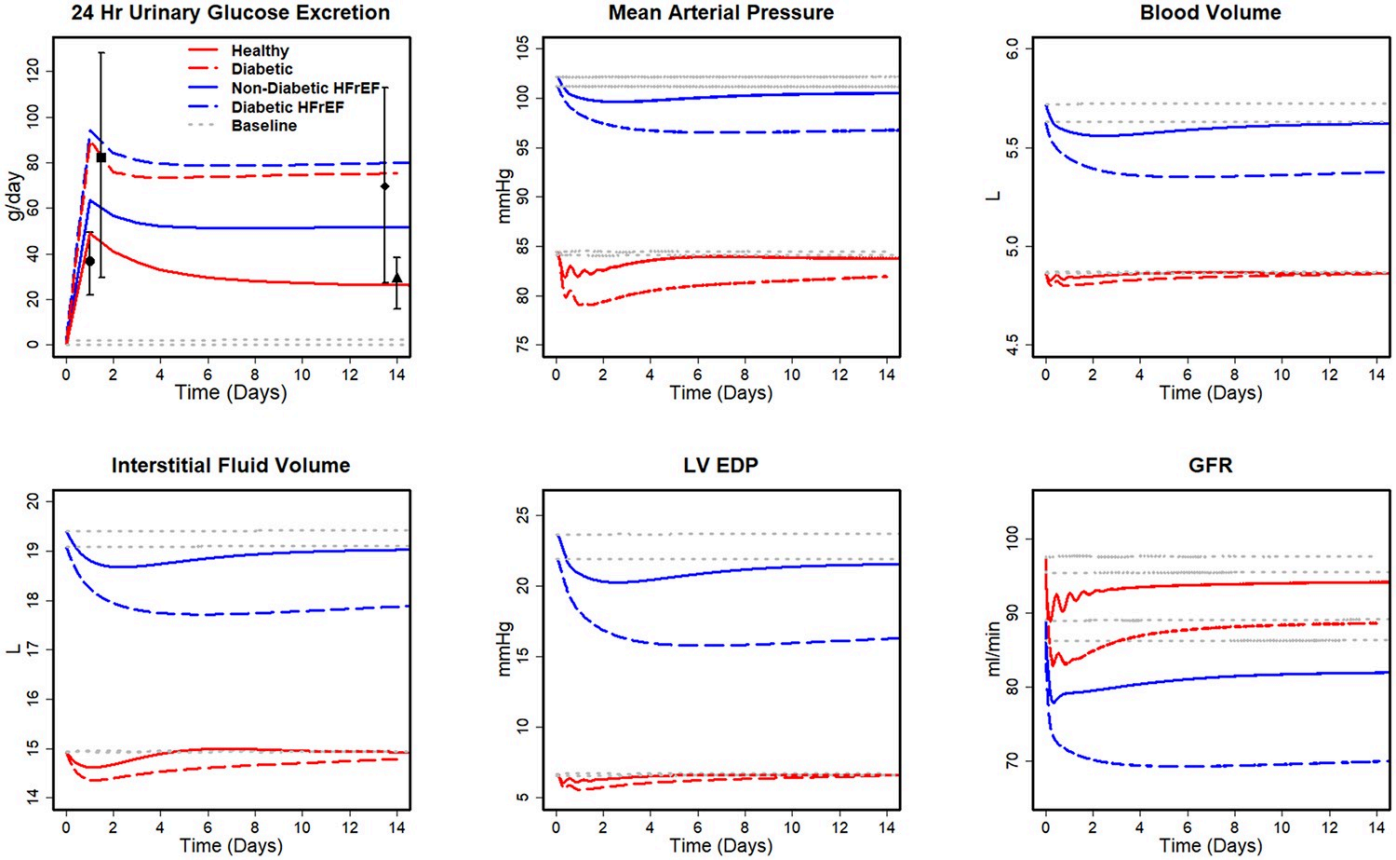
Simulated physiological responses to SGLT2i treatment for healthy, diabetic, Non-diabetic HF-rEF, and diabetic HF-rEF subjects. Square (■) and diamond (♦) are the total amount of UGE on days 1 and 14 by type 2 diabetes mellitus patients reported in [58] Circle (●) and triangle (▲) are the total amount of UGE on days 1 and 14 by healthy subjects reported in [35].

The cardiac hemodynamic responses to SGLT2i were simulated in four virtual patients: healthy, diabetes, HF-rEF with diabetes, and HF-rEF without diabetes. Fig 3 shows the time course of the predicted hemodynamic response to SGLT2i during the first two weeks. The simulated changes in 24hr UGE are in agreement with clinical data [35]. Glucose excretion in the non-HF-rEF diabetic is about two times larger than that of a healthy subject. UGE is similarly increased in diabetic HF-rEF subjects compared to non-diabetic HF-rEF subjects. MAP decreased in all the four cases by 3–5 mmHg, consistent with reported levels of the reduction in blood pressure with SGLT2i [54–57]. Simulations also predict reductions in BV and IFV, with much larger reductions in IFV relative to BV, as we have predicted previously for subjects without HF [35].

The predicted reduction in BV and IFV is larger in HF-rEF than in non-HF-rEF (Fig 3). Volumes are reduced in both diabetic (BV: 1.6%, IFV: 4.1%) and non-diabetic HF-rEF (BV: 1.5%, IFV: 6.1%), although the reductions are substantially larger in HF-rEF with diabetes than in HF-rEF without diabetes (BV: 3.3%, IFV: 16.7%). The differential effect of SGLT2i in reducing IFV relative to BV previously predicted in healthy subjects is sustained in HF-rEF.

SGLT2i is predicted to decrease LVEDP by 4.2 and 1.9 mmHg in HF-rEF patients with and without diabetes, respectively. On the other hand, in patients without HF (and thus without elevated LVEDP at baseline), the decrease in LVEDP is much smaller and transient, quickly returning to baseline. In both patients with and without HF-rEF, SGLT2i causes an initial decrease in GFR, and the reduction is smaller in non-diabetics than in diabetics, regardless of HF status.

**Fig 4:**
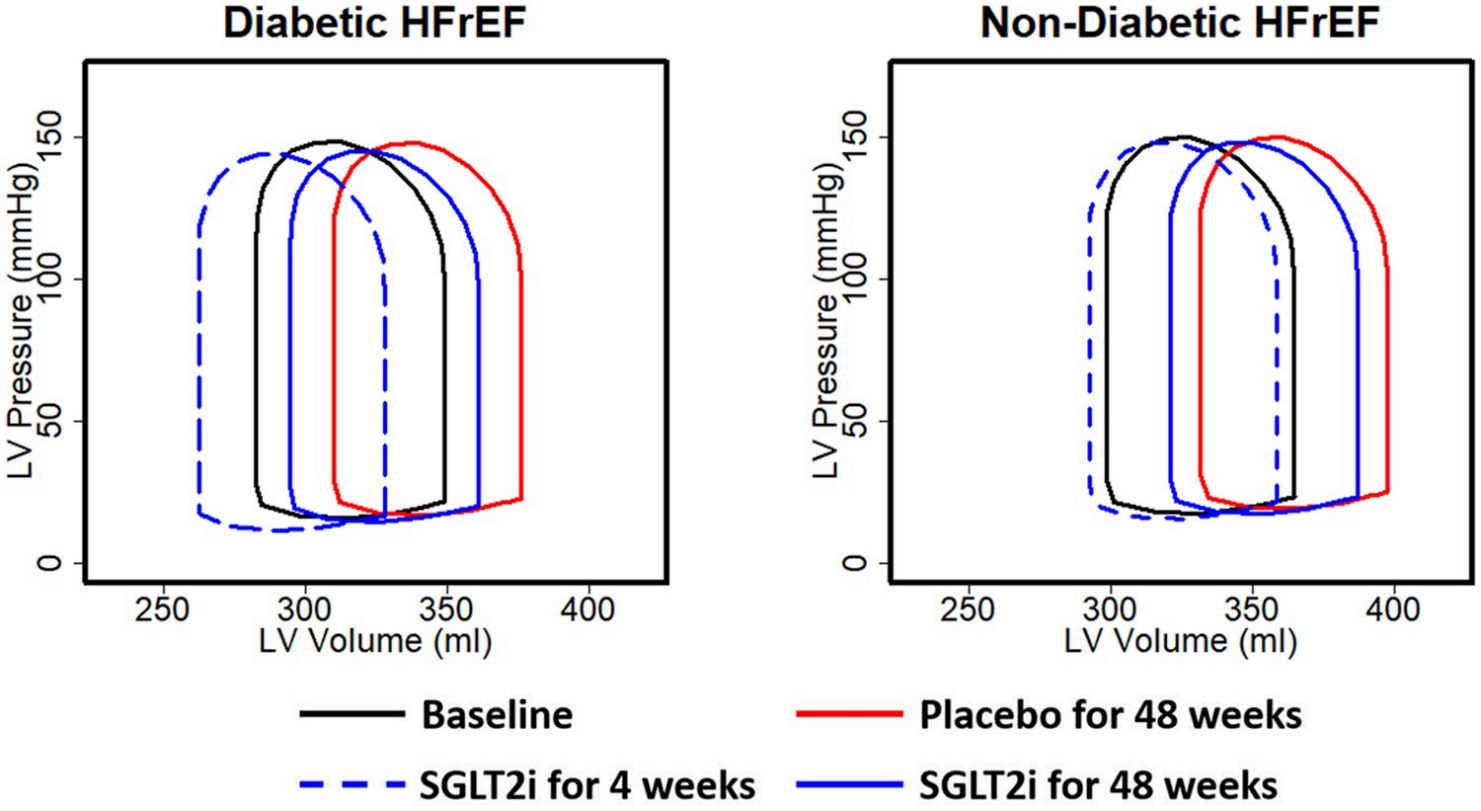
Simulated effect of SGLT2i on pressure-volume loops in HF-rEF, with (left) and without (right) diabetes, acutely (4 weeks) and after 48 weeks.

Fig 4 shows the simulated effect of SGLT2i on the P-V loop progression in non-diabetic HF-rEF and diabetic HF-rEF after 4 and 48 weeks of treatment. As in the SOLVD study (Fig 1), the P-V loops for the placebo group shifted to the right over time (Fig 4 solid black vs. red loops). With 4 weeks of SGLT2i, the P-V loop is predicted to shift leftward (blue dashed vs. black loops), due to hemodynamic unloading of the heart in response to BV and MAP reductions by SGLT2i. This leftward shift occurred because SGLT2i reduced LVEDP (Fig 3), alleviating excess preload. Because the LVEDP reduction was larger in diabetics than non-diabetics, the leftward shift of the P-V loop was greater in diabetic HF-rEF (Fig 4 left vs. right). Since LVEDP is reduced not completely normalized, volume overload on the heart is reduced but not eliminated. Thus, the P-V loop progresses to the right between weeks 4 and week 48 (dashed blue vs. solid blue loops) but, this progression occurs at a slower rate than in the placebo, due to the lower LVEDP. By 48 weeks of treatment, the P-V loops in the SGLT2i virtual patients remain substantially leftward shifted relative to placebo in both diabetics and non-diabetics (solid blue vs. solid red loops).

## Discussion

In this study, an integrated model of cardiorenal function was updated and utilized to simulate states of HF-rEF. After matching an HF-rEF virtual patient to the baseline hemodynamic characteristics in the SOLVD clinical trial [42], the model was able to describe the rightward progression of the P-V loop in the placebo arm, as well as the leftward shift in the enalapril arm, providing validation that the model adequately describes the progression of cardiac remodeling in response to hemodynamic overload in HF, as well as improvements with renally acting therapies. The model was then applied to investigate cardiac hemodynamic and systemic volume responses that may explain clinical observed improvements in HF outcomes with SGLT2i. The model predicts that SGLT2i reduces LVEDP in both diabetic and non-diabetic HF-rEF (although reductions are larger in diabetics), and that this preload reduction slows cardiac remodeling and progression over one year. It further predicts that SGLT2i will reduce interstitial congestion without inducing large reductions in blood volume.

### Simulating progression of HF-rEF

The model was able to reproduce a state with the characteristics of HF-rEF, including suppressed ejection fraction as well as increased LVEDP and IFV reflective of volume overload and congestion. This state might be considered representative of a patient who has previously experienced a myocardial infarction (MI) or other ischemic damage but is now in a stable post-MI state. We do not attempt to account for the acute changes immediately after ischemia, but only the resulting consequences once myocyte loss and cardiac dilatation have occurred. In this analysis, we focused on the hemodynamic state of HF-rEF at rest, and we did not consider aspects of HF associated with the reduced cardiac reserve in an impaired activity. The model was also able to describe the rate of progressive cardiac remodeling reflected by the rightward shift in the P-V loop in the placebo arm of the SOLVD clinical study over one year. Coupled with our previous study [40], the framework first proposed by Grossman [38], relating LV peak systolic stress and LV end-diastolic stress to cardiac remodeling through increases in myocyte length and diameter, respectively, is sufficient to represent remodeling patterns and time courses in a variety of cardiac dysfunctions, including aortic stenosis, mitral regurgitation, hypertension, and now HF-rEF.

### Simulating the effect of ACE inhibition on cardiac hemodynamics in HF-rEF

Treatment with an ACE inhibitor is part of the standard of care treatment for HF-rEF, and the SOLVD study was one of the first studies to demonstrate the effect of ACEi on HF morbidity and mortality. Thus, the cardiac hemodynamic effects observed in the SOLVD cardiac hemodynamic substudy simulated here demonstrate that these benefits were due at least in part to improvements in cardiac hemodynamics with ACEi. While the model time constants were specifically calibrated to match the placebo P-V loop progression in SOLVD, the ability of the model to predict the leftward shift of the P-V loop as well as reductions in LV EDP in the enalapril-treated arm, without further parameter changes, provides validation that the model reasonably describes the effects of hemodynamic loading on cardiac remodeling in HF-rEF. It also further demonstrates the model’s ability to describe the consequences of renal therapeutic targets on cardiac functions. We have previously shown that the renal vasodilatory and natriuretic effects of enalapril were sufficient to describe its antihypertensive effects [30]. Here we further demonstrate that these renal mechanisms are able to explain its effects on HF-rEF cardiac functions and remodeling.

### Prospective simulation of the effect of SGLT2i on cardiac hemodynamics in HF-rEF

The cardiorenal model was used to prospectively simulate the hemodynamic response to dapagliflozin treatment in diabetic and non-diabetic virtual patients. Agreements between the simulated and measured 24hr UGE in healthy and diabetic subjects [18,39] indicate that the appropriate pharmacodynamic effect of dapagliflozin on proximal tubule SGLT2i is represented in the model. The predicted modest reductions in mean arterial pressure in non-HF patients of 2–5 mmHg are consistent with clinically reported values [54,57]. Predicted MAP changes in HF-rEF patients were similarly modest. This may be clinically important, since most current standard-of-care therapies for HF-rEF are antihypertensive therapies, and may increase the risk for hypotension [59].

While the DAPAHF study convincingly demonstrated that SGLT2i reduces HF morbidity and mortality, to our knowledge, no mechanistic studies have yet been reported to investigate how SGLT2i impacts cardiac hemodynamics. These simulations help fill that gap. The simulations predict that dapagliflozin reduces LV EDP and shifts the P-V loop to the left the initial weeks of treatment. Because LVEDP is not normalized, remodeling is slowed but not completely arrested. Progression is slowed relative to placebo in both the diabetic and non-diabetic. However, because the predicted reductions in LVEDP are greater in the diabetic patient than in the nondiabetic, the initial leftward shift is greater, and the rate of progression is slowed more.

The predicted improvements in cardiac hemodynamics with SGLT2i are a consequence of its natriuretic and diuretic effects, which result in reductions in BV and IFV. In previous simulations of diabetics without HF, we modeled and described in detail the ways in which the osmotic diuresis effect of SGLT2i results in larger reductions in IFV compared to BV. Briefly, we showed mathematically that several mechanisms likely work together to produce the clinically observed response to SGLT2i. Reduced reabsorption of sodium, both through direct inhibition of SGLT2 and indirect inhibition of NHE3, as well as the osmotic diuretic effect of glucose remaining in the tubules, results in excess electrolyte-free water clearance. Subsequently, redistribution of sodium between the blood, interstitium, and nonosmoticaly bound sodium in peripheral tissues results in a shift in fluid out of the interstitium to compensate for the loss of electrolyte-free water [60].

Accumulation of IFV is one of the primary causes of congestion in the body. It was often observed the occurrence of a ‘drowning sensation’ when the heart is failing in a subject as a consequence of pulmonary edema. Thus, we speculated that these effects potentially attenuate congestion and edema without excessive reductions in plasma volume and may explain the benefits in HF observed in the EMPA-REG, CANVAS, and DECLARE studies. These studies consisted of T2D patients with increased cardiovascular risk, as opposed to patients with overt HF at baseline. The DAPAHF study confirmed that the benefits observed in these studies extent to patients with overt HF-rEF [10]. Our current simulations showing that the volume effects of SGLT2i are expected to be even more pronounced in HF-rEF, and that the relatively greater clearance from the interstitium compared to blood volume is maintained.

### Limitations

One major limitation of the cardiorenal model is that RAAS pathophysiological effect has not been validated through a clinical trial with given the baseline characteristics of patients even though the progression of HF-rEF over time reported in SOLVD study can be captured. It is suggested that further studies on the hemodynamic alterations in HF-rEF patients with SGLT2i would be required to confirm the current findings. Sex-specific differences in physiology and pathophysiology were not considered in the simulation of HF-rEF [33]. HF-rEF is more prevalent in men [61,62], and most studies of HF-rEF have included predominantly male populations, and thus model parameters likely reflect physiology more representative of males. Differences in females could impact simulated renal and cardiac responses to drug treatments.

For validating the pathological interactions between the heart and kidney ACEi enalapril was assumed to only have a natriuretic effect on the kidney in promoting water and sodium excretions. Effects of ACEi on cardiac functions, nervous activities, and vascular functions were not taken into account, as a result LV pressure continues to decrease over time as observed in the placebo arm. The reductions in blood and interstitial fluid volume after ACEi lead to further decreases in LV pressure, indicating an overall downward shifted P-V loop.

Cardiac hemodynamics were simulated using a simple lumped parameter model of the circulation, and does not account for temporal and special complexities of aortic flow, wave reflection from the peripheral sites and propagation of wave velocity, which likely affect the speed of ejection during early systole. In addition, the shape of the heart was considered as a sphere and cardiac valves were assumed to be perfect valves. Consequently, these simplifications may contribute to differences in the shape of the P-V loop compared to the clinical data in SOLVD, such as the over-prediction of the speed of ejection in early systole (Fig 1).

SGLT2i may involve in the activation of the renal sympathetic nerve activity and SGLT2i may attenuate the renal afferent nervous activity [63]. SGLT2i was also found to be associated with the reduction in the heart rate in HF-rEF patients [64]. However, these protecting effects were not considered in the current analysis.

The current analysis simulates a single representative virtual patient and thus cannot account for variability in clinical response by simulating one HF-rEF virtual patient. This is sufficient for the goals of this study, including demonstrating that the integrated cardiorenal model reproduces states of heart failure, the progression of heart failure, and the hemodynamic improvement and remodeling response to treatment. However, future work producing HF-rEF virtual patients with variation in underlying pathophysiological parameters may allow the evaluation of the variability in disease progression and regression with treatment.

Last but not the least, when simulating the state of diabetes, the damage to renal functions due to diabetes, such as the loss of nephrons, were not considered, and the elevation in arterial pressure due to diabetes-induced systemic arteriosclerosis was not considered for simplicity [65]. Despite the limitations, the current model can be extended to establish a “fit for purpose” design to study physiology and pharmacology.

## Conclusions

The current study proposed a cardiorenal mathematical model to study the pathophysiological interplay between the heart and kidney by conjugating existing cardiac and renal models. The cardiorenal model is able to reproduce the state and progression of HF-rEF, and improvements in HF-rEF with ACEi as reported in the SOLVD clinical trial. The cardiorenal model was used to evaluate the effect of SGLT2i on cardiac functions, exhibiting reductions in IFV, BV, and LVEDP, potentially decreasing congestion and edema in HF-rEF patients.

## Materials and methods

### Mathematical model

**Fig 5:**
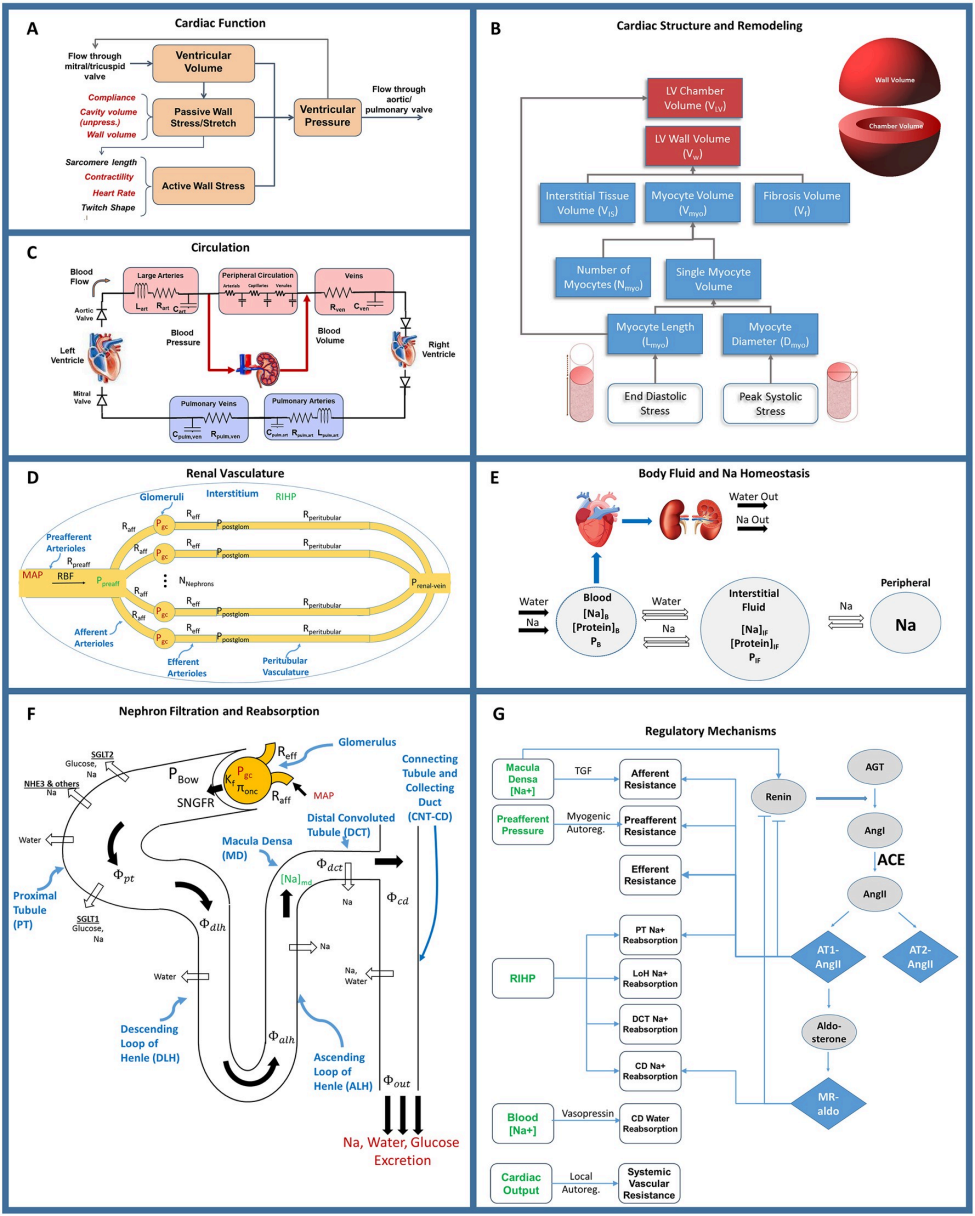
The integrated cardiorenal model links cardiac mechanics. Ventricle simulation and remodeling (A) and (B), a lumped parameter description of cardiovascular circulation (C), whole body Na+ and fluid homeostasis (E), renal hemodynamics (D), renal filtration and reabsorption (F), and neurohormonal and intrinsic feedbacks including the renin-angiotensin-aldosterone system (RAAS) (G).

We utilized a previously published model of the cardiorenal function [40]. Model equations added or altered from previously published forms, or critical to understanding the current results, are described here. Full model equations, parameters, and initial conditions, as well as the results of validation tests confirming appropriately model behavior, can be found in the supplemental material [17,66]. The major functional modules and mechanisms of the mathematical model are shown schematically in Fig 5. We previously extended a dynamic model of cardiac ventricular function developed by Bovendeerd et al (Fig 5A and 5C) to account for adaptation of myocytes and remodeling of the LV in response to changes in mechanical loading (Fig 5B). This model was also integrated with our previously published model of renal function and volume homeostasis (Fig 5D–5G), to allow for interaction between cardiac and renal functions [30,35,67]. The renal and cardiac portions of the model are coupled through 1) blood volume, which is regulated by kidneys through control of sodium and water excretion (Fig 5E and 5F), and is a key determinant of blood pressure in the circulation (Fig 5C), and 2) mean arterial pressure (time-averaged arterial blood pressure), which is calculated in the circulation sub-model (Fig 5C) and is a key determinant of renal perfusion and glomerular filtration rate in the kidney model (Fig 5D and 5F).

### Modeling cardiac remodeling

Cardiac myocyte hypertrophy is an adaptive response of the myocardium to changing loading conditions, leading to an increase in the myocyte length and/or myocyte diameter. Grossman et al suggested that chronically elevated peak LV systolic wall stress (pressure overload) stimulates the parallel addition of contractile sarcomere units as a compensatory mechanism [68]. This increases individual myocyte cross-sectional area without a significant change in length. On a macroscopic level, the result is increased LV wall thickness (concentric remodeling) [69,70]. In conditions of volume overload, when the diastolic LV wall stress is increased beyond normal values, the sarcomeres are added predominantly in series with the corresponding increase in myocytes’ length and, consequently, LV cavity diameter (eccentric remodeling) [69,70].

To describe these remodeling phenomena, the LV chamber wall volume *V*_*w*_ was modeled as the sum of the volume of myocytes (*V*_*myo*_), interstitial tissue (*V*_*IS*_), and fibrosis (*V*_*f*_).

$$V_{W}=V_{myo}+V_{IS}+V_{f}$$ (1)

Myocytes were modeled as cylinders, and myocyte volume was determined as

$$V_{myo}=N_{myo}(\frac{\pi D_{myo}^{2}L_{myo}}{4})$$ (2)

where *N*_*myo*,_ *D*_*myo*,_ *L*_*myo*_ are the number, average diameter, and average length of myocytes. *D*_*myo*_ is the sum of the normal (healthy) diameter *D*_*myo*,*0*_ and any change in diameter Δ*D*_*myo*_ resulting from remodeling. *L*_*myo*_ is the sum of normal (healthy) length *L*_*myo*,*0*_ and any change in length Δ*L*_*myo*_.

$$D_{myo}=D_{myo,0}+\Delta D_{myo}$$ (3)

$$L_{myo}=L_{myo,0}+\Delta L_{myo}$$ (4)

As proposed by Grossman, we assumed that the heart remodels to maintain a certain level of peak elastic stress *σ*_*f*_ in the cardiac tissue fiber. Simulation of fiber stresses, as developed by Bovendeerd et al, is described in detail elsewhere [17,66]. The peak systolic stress *σ*_*f*,*peak*,*0*_ under baseline conditions is used as the reference stress level. When the peak stress along the fiber *σ*_*f*,*peak*_ is greater than *σ*_*f*,*peak*,*0*_, myocyte diameter increases, representing the formation of sarcomeres in parallel. When *σ*_*f*,*peak*_ is less than *σ*_*f*,*peak*,*0*_, myocyte diameter decreases.

$$\frac{d({\Delta D}_{myo})}{dt}=K_{d}\left(\frac{\sigma_{f,peak}}{\sigma_{f,peak,0}}-1\right)$$ (5)

Myocytes cannot increase in diameter infinitely. As the diameter approaches the maximum value, the rate constant for the increase in diameter approaches zero:

$$K_{d}=\left\{ \begin{array}{ll} K_{d0}*\frac{\Delta D_{max}-\Delta D}{\Delta D_{max}}, & \sigma_{f,peak}\geq \sigma_{f,peak,0}\\ K_{d0} & \sigma_{f,peak}<\sigma_{f,peak,0} \end{array}\right.$$ (6)

Δ*D*_*max*_ is assumed to be equal to *D*_*myo*,*0*_, so that the increase in diameter is limited to 2X normal.

Similarly, we assume that myocytes add sarcomeres in series and thus increase in length when LV passive stress along the fiber at the end of diastole is elevated. LV end-diastolic (ED) stress under baseline conditions is used as the reference stress level *σ*_*f*,*ED*,*0*_. When the ED passive stress along the fiber *σ*_*f*,*ED*_ is greater than *σ*_*f*,*ED*,*0*_, myocyte length increases, representing the formation of sarcomeres in series.

$$\frac{d(\Delta L)}{dt}=K_{l}(\frac{\sigma_{f,ED}}{\sigma_{f,ED0}}-1)$$ (7)

Kl={Kl0*ΔLmax-ΔLΔLmaxσf,ED≥σf,ED,00σf,ED<σf,ED,0 (8)

Δ*L*_*max*_ is assumed to be equal to *L*_*myo*,*0*_, so that the increase in length is limited to 2X normal.

The left ventricle cavity volume at zero transmural pressure increases as the myocyte length increases, according to the equation

$$V_{LV,cavity}=V_{LV0}{\left(1+L_{scale}\frac{\Delta L}{L_{myo,0}}\right)}^{3}$$ (9)

where *V*_*LV0*_ is the cavity volume before any remodeling has occurred and *L*_*scale*_ is a coefficient for the approximation and taken to be 1 in this case.

These responses are governed by rate constants *K*_*l0*_ and *K*_*d0*_. These rate constants were previously estimated based on the time course of hypertrophy regression with antihypertensive therapy [40]. Here, we further refined this calibration by fitting the progression of the P-V loop in the placebo arm of the SOLVD hemodynamic substudy[42].

### Modeling starling forces in capillary and interstitial fluid exchange

Accumulation of edematous fluid is a salient feature of HF, and thus the model was updated to fully describe the mechanisms determining fluid movement between compartments. Movement of fluid between the blood and interstitium is governed by starling forces between capillary vessels and the interstitium

$$\Phi_{FR}=K_{f}(P_{c}-P_{if}-\pi_{protein,c}+\pi_{protein,if}-\pi_{Na,c}+\pi_{Na,if})$$ (10)

Here, *Φ*_*FR*_ is the capillary filtration rate; *K*_*f*_ is the capillary filtration coefficient [71]; Subscripts *c* and *if* represent the capillary system and interstitium, respectively; *P* is the hydrostatic pressure, *π*_*protein*_ is the colloid osmotic pressure; *π*_*NA*_ is the osmotic pressure due to sodium.

**Fig 6:**
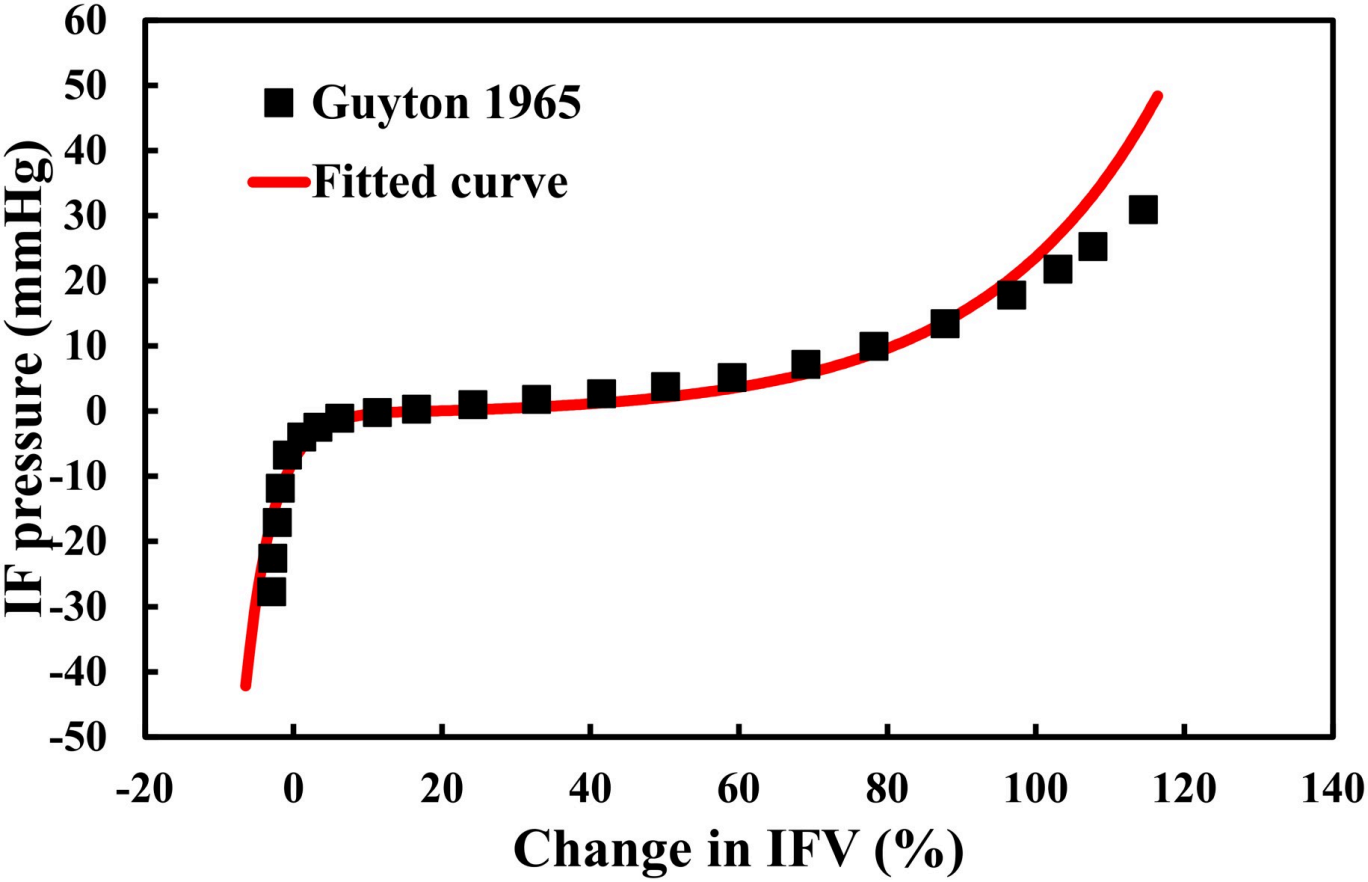
The relationship between interstitial fluid volume and pressure. The continuous line represents the fitted polynomial function based on the data [72].

In the absence of HF, capillary pressure is normally well-controlled, and thus the model previously only accounted for the role of osmotic pressure. Here, we adapted the model to incorporate the role of changes in hydrostatic pressure. The mean capillary hydrostatic pressure was calculated from the pressure waveform obtained by solving the lumped vascular system in the cardiac model. The relationship between interstitial pressure and IFV was determined by fitting the measured relationship in [72], as shown in Fig 6. Since the interstitium is a non-linear strain-stiffening material, the pressure-volume relationship was expressed with different exponential functions in states of volume expansion and contraction.

$$P_{if,expansion}=A_{if,ex}\text{exp}\left(B_{if,ex}\times IFV\right)-C_{if,ex},(for\;IFV\geq 15)$$ (11)

$$P_{if,contraction}=-\frac{A_{if,con}}{\text{exp}\left(B_{if,con}\times (IFV-12)\right)}+C_{if,con},(for\;IFV<15)$$ (12)

Colloid osmotic pressure was determined according to the Landis-Pappenheimer equation [18,73]:

$$\pi_{protein}=1.629\times C_{prot,i}+0.2935\times C_{prot,i}^{2}$$ (13)

Here, *C*_*prot*_ is the plasma protein concentration; subscript *i* is the blood or interstitial compartment. The amount of protein in the blood and interstitium was assumed constant so that the concentration depends only on the volume of fluid in the compartment.

The osmotic pressure due to sodium was determined as:

$$\pi_{Na}=C_{Na,i}RT$$ (14)

where *C*_*Na*_ is the sodium concentration; *R* is the ideal gas constant; *T* is the absolute temperature. The reference model parameters are shown in Table 3.

**Table 3:** Reference settings for parameters in the model.

Parameters	Definitions	Values	Unties
K_f_	Capillary filtration coefficient	6.67	ml·min^-1^·mmHg^-1^ [71]
K_l0_	Rate constant for myocyte length	1E-10	-
K_d0_	Rate constant for myocyte diameter	1.33E-11	-
*A*_*if*,*ex*_	Coefficient in the pressure-volume relationship during expansion	0.01	-
*B*_*if*,*ex*_		0.25	mmHg/L
*C*_*if*,*ex*_		-0.72	mmHg
*A*_*if*,*con*_	Coefficient in the pressure-volume relationship during contraction	-199	-
*B*_*if*,*con*_		1.55	mmHg/L
*C*_*if*,*con*_		0.079	mmHg
*R*	Ideal gas constant	8.314	J⋅K^−1^⋅mol^−1^
*T*	Absolute temperature	310	K

Model Qualification: After altering the model, we confirmed that the baseline behavior was stable, that all feedback mechanisms were at their setpoint in the base case, and the model remained consistent with previously demonstrated responses to therapies. We confirmed that the model still reproduces that change in plasma renin concentration and mean arterial pressure observed with antihypertensive therapies targeting on various components in RAAS, as reported in [30], and that produces urinary and plasma biomarker responses to SGLT2 inhibition as reported in [35]. See supplement S1–S3 Figs.

### Summary of SOLVD study

Briefly, the SOLVD clinical trial evaluated the effect of ACE inhibitor enalapril on morbidity and mortality in a population of HF-rEF patients. In the clinical trial, 56 patients were enrolled in a radionuclide substudy, and of these, 16 also participated in a catheterization substudy. Patients were randomized to receive enalapril or placebo, and radionuclide and catheterization studies were performed before and 1 year after randomization. Baseline functional measures of all substudy patients, as well as P-V loop data from the catheterization substudy, were used for model calibration and validation in the current analysis.

## Supporting information

S1 Text Full model equations. (DOCX)

S1 Fig At baseline, feedback mechanisms in the model are stable and at their setpoint value. Myocyte diameter and length are also at their baseline value and are unchanging, indicating that LV peak systolic stress and LV end diastolic stress are at or below threshold levels for remodeling.
(TIF)

S2 Fig Validation test for ability of model to simulation response to antihypertensive therapies. Effects of antihypertensive therapies (aliskiren [Ali], valsartan [Val], candesartan [cand], enalapril [enal], eplerenone [epl], irbesartan [irb], losartan [los], ramapril [ram] were previously calibrated and validated in the renal-only version of this model. As validation of the integrated model, we repeated these simulations, and show here that the integrated model produces changes in plasma renin concentration (a) and mean arterial pressure (b) consistent with clinically observed levels.
(TIF)

S3 Fig Validation test for ability of model to simulation response to dapagliflozin for healthy subjects. As validation of the integrated model, we repeated these simulations, and show here that the integrated model produces changes in urinary glucose excretion (a), water excretion (b), mean arterial pressure (b), sodium excretion (c), serum creatinine (d), and plasma sodium concentration (E) consistent with clinically observed levels responses.
(TIF)

S1 Table Additional cardiac model parameters. (DOCX)

S2 Table Circulatory model parameters. (DOCX)

S3 Table Renal model parameters. (DOCX)

S4 Table Regulatory mechanisms model parameters. (DOCX)

S5 Table Renin angiotensin aldosterone system model parameters. (DOCX)

S6 Table Model initial conditions. (DOCX)

S1 Appendix The derivation of Equation S69. (DOCX)
