# Supplementary figures and images for: Cardiac and renal function interactions in heart failure with reduced ejection fraction: A mathematical modeling analysis
Source: PLoS Comput Biol. 2020 Aug 17;16(8):e1008074. doi: 10.1371/journal.pcbi.1008074 (PMC7451992; doi:10.1371/journal.pcbi.1008074)

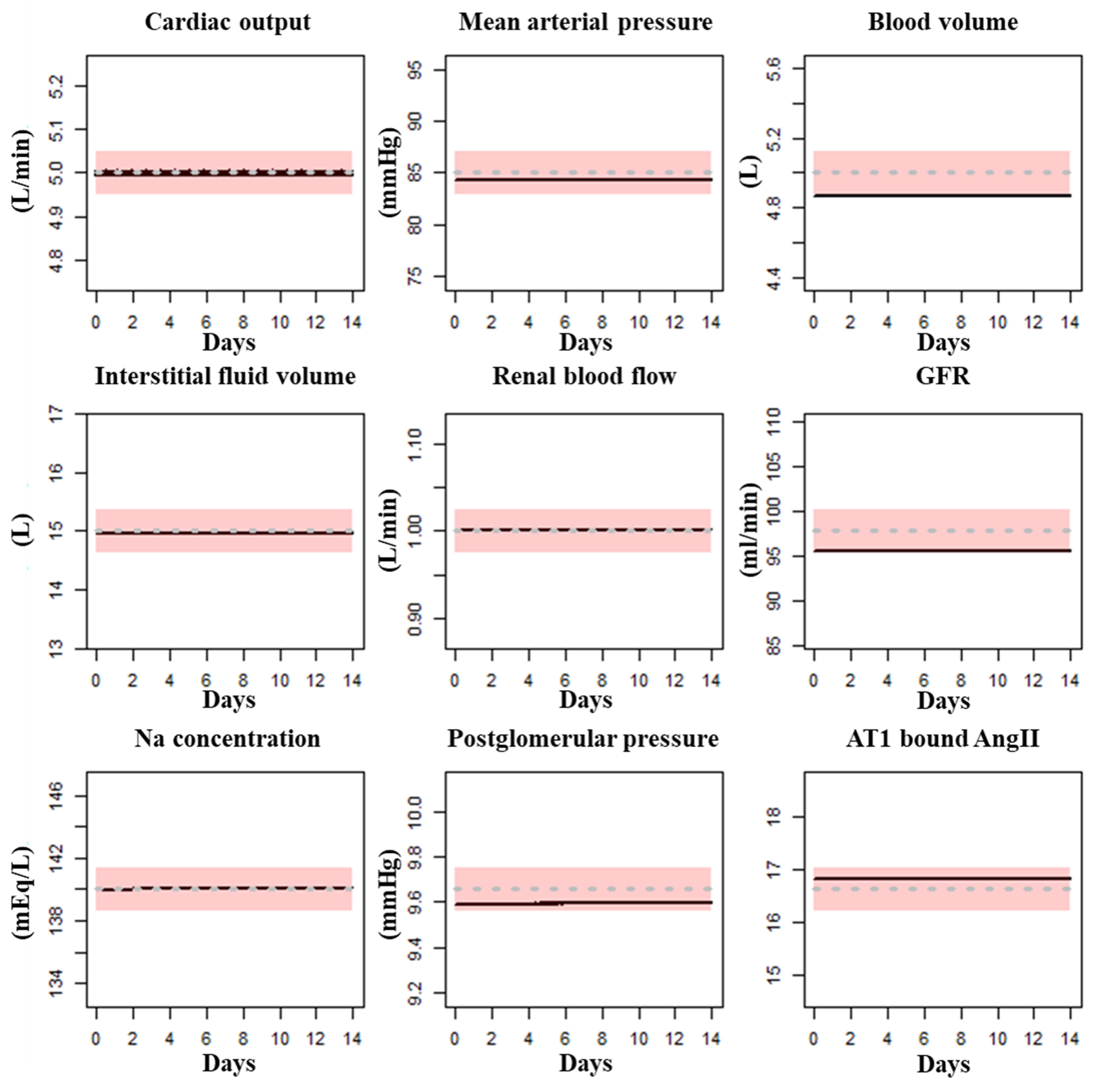

Supplement: S1 Fig — Myocyte diameter and length are also at their baseline value and are unchanging, indicating that LV peak systolic stress and LV end diastolic stress are at or below threshold levels for remodeling. (TIF) [file pcbi.1008074.s002.tif]

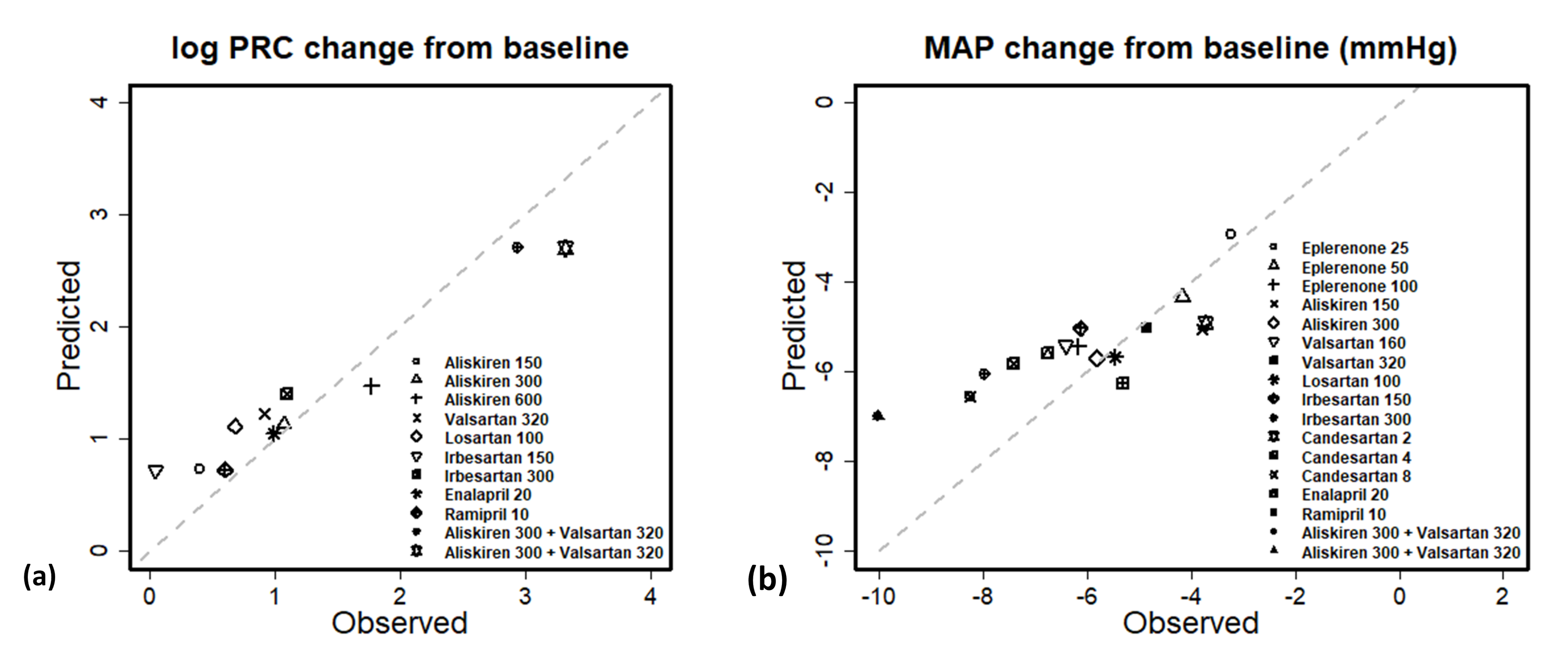

Supplement: S2 Fig — Effects of antihypertensive therapies (aliskiren [Ali], valsartan [Val], candesartan [cand], enalapril [enal], eplerenone [epl], irbesartan [irb], losartan [los], ramapril [ram] were previously calibrated and validated in the renal-only version of this model. As validation of the integrated model, we repeated these simulations, and show here that the integrated model produces changes in plasma renin concentration (a) and mean arterial pressure (b) consistent with clinically observed levels. (TIF) [file pcbi.1008074.s003.tif]

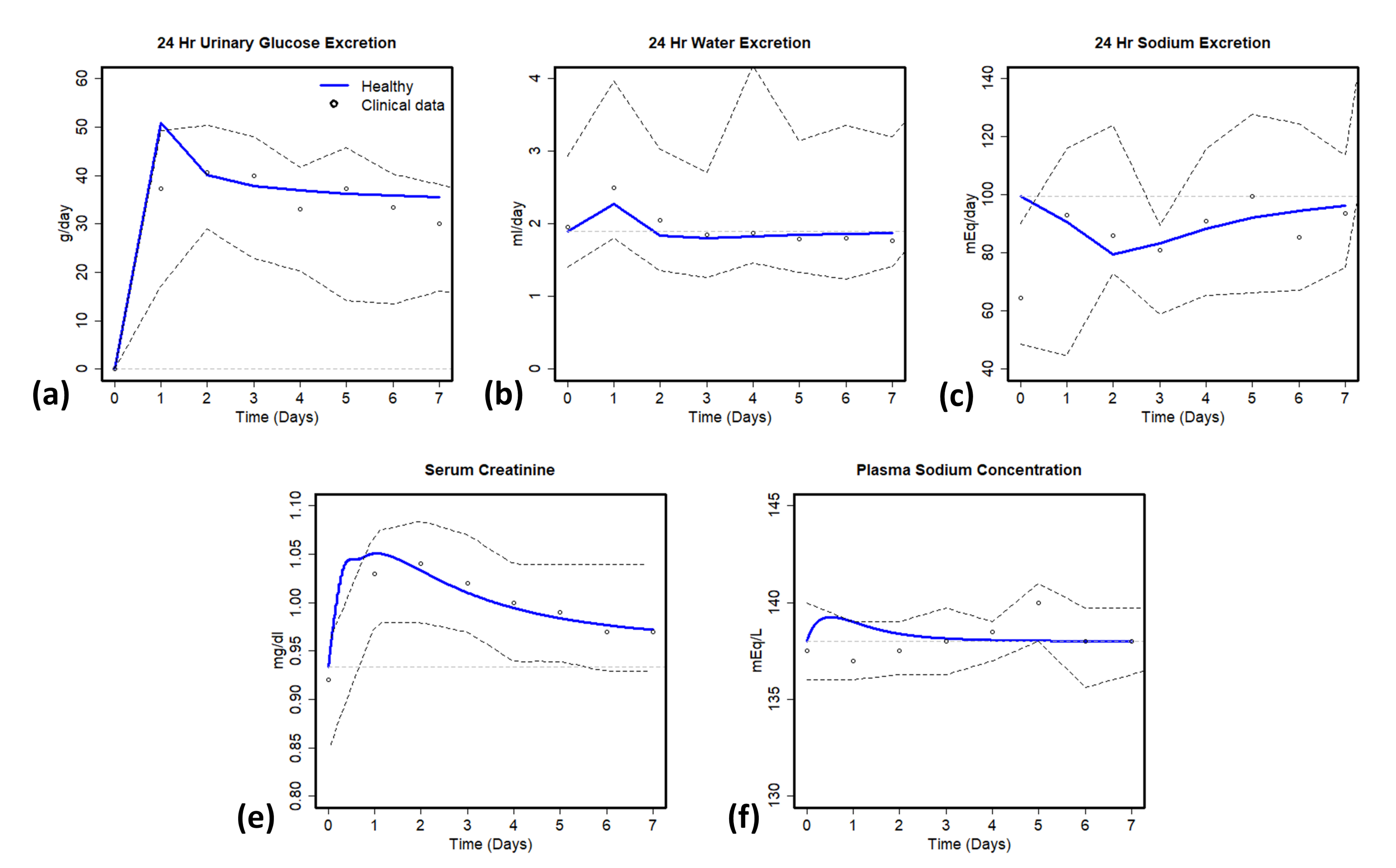

Supplement: S3 Fig — As validation of the integrated model, we repeated these simulations, and show here that the integrated model produces changes in urinary glucose excretion (a), water excretion (b), mean arterial pressure (b), sodium excretion (c), serum creatinine (d), and plasma sodium concentration (E) consistent with clinically observed levels responses. (TIF) [file pcbi.1008074.s004.tif]
