# Supplementary material for: Cardiac and renal function interactions in heart failure with reduced ejection fraction: A mathematical modeling analysis
Source: PLoS Comput Biol. 2020 Aug 17;16(8):e1008074. doi: 10.1371/journal.pcbi.1008074 (PMC7451992; doi:10.1371/journal.pcbi.1008074)
Supplement: S1 Table — (DOCX) [file pcbi.1008074.s005.docx]

| **Parameter** | **Definition** | **Value** | **Units** |
| --- | --- | --- | --- |
| C | cardiac contractility | 1 | - |
| c_f_ | LV stiffness along the fiber | 11 | - |
| c_r_ | LV radial stiffness | 9 | - |
| C_f,RV_ | RV stiffness along the fiber | 9 | - |
| C_r,RV_ | RV radial stiffness | 9 | - |
| l_s,a0_ | Sarcomere length below which active stress becomes zero | 1.9 | µm |
| l_s,ar_ | Sarcomere length to which the reference stress σ_ar_ is referenced to | 2 | µm |
| σ_ar_ | Active stress scaling constant | 55 | kPa |
| σ_f0_ | Longitudinal passive stress at zero stretch | 0.9 | kPa |
| σ_r0_ | Radial passive stress at zero stretch | 0.2 | kPa |
| v_0_ | Unloaded sarcomere shortening velocity | 50 | µm/s |
| V_w,rv_ | RV wall volume | 100 | mL |
