# Supplementary material for: Cardiac and renal function interactions in heart failure with reduced ejection fraction: A mathematical modeling analysis
Source: PLoS Comput Biol. 2020 Aug 17;16(8):e1008074. doi: 10.1371/journal.pcbi.1008074 (PMC7451992; doi:10.1371/journal.pcbi.1008074)
Supplement: S2 Table — (DOCX) [file pcbi.1008074.s006.docx]

| **Parameter** | **Definition** | **Value** | **Units** |
| --- | --- | --- | --- |
| R_art0_ | Systemic resistance | 5e6 | Pa-s/m^3^ |
| R_als0_ | Arterials resistance | 8.5e7 | Pa-s/m^3^ |
| R_caps0_ | Capillaries resistance | 3.17e7 | Pa-s/m^3^ |
| R_vns0_ | venules resistance | 1.12e7 | Pa-s/m^3^ |
| R_ven_ | venous resistance | 5e6 | Pa-s/m^3^ |
| R_mitral_ | Mitral valve resistance | 1e6 | Pa-s/m^3^ |
| R_pulm,art_ | pulmonary arterial resistance | 3e6 | Pa-s/m^3^ |
| R_pulm,ven_ | pulmonary venous resistance | 6.4e6 | Pa-s/m^3^ |
| R_RA_ | right atrial resistance | 1e6 | Pa-s/m^3^ |
| V_art0_ | arterial volume at zero transmural pressure | 450 | mL |
| V_per0_ | peripheral volume at zero transmural pressure | 420 | mL |
| V_ven0_ | venous volume at zero transmural pressure | 3000 | mL |
| V_pulm,art0_ | pulmonary arterial volume at zero transmural pressure | 40 | mL |
| V_pulm,ven0_ | pulmonary venous volume at zero transmural pressure | 250 | mL |
| V_RV0_ | right ventricle volume at zero transmural pressure | 75 | mL |
| V_w,RV_ | right ventricle wall volume | 100 | mL |
| C_art_ | arterial compliance | 1.1e-8 | m^3^/Pa |
| C_per_ | peripheral vascular compliance | 1e-8 | m^3^/Pa |
| C_ven_ | venous compliance | 1.8e-7 | m^3^/Pa |
| C_art,pulm_ | arterial compliance | 2e-8 | m^3^/Pa |
| C_per,ven_ | peripheral vascular compliance | 1.65e-7 | m^3^/Pa |
| I_art_ | arterial **inertance** | 60 | kPa-s/m^3^ |
| I_pulm_ | pulmonary arterial **inertance** | 60 | kPa-s/m^3^ |
