# Supplementary material for: Cardiac and renal function interactions in heart failure with reduced ejection fraction: A mathematical modeling analysis
Source: PLoS Comput Biol. 2020 Aug 17;16(8):e1008074. doi: 10.1371/journal.pcbi.1008074 (PMC7451992; doi:10.1371/journal.pcbi.1008074)
Supplement: S4 Table — (DOCX) [file pcbi.1008074.s008.docx]

| **Parameter** | **Definition** | **Value** | **Units** |
| --- | --- | --- | --- |
| Φ_Na,md,0_ | Setpoint for sodium flow delivered to the macula densa | 0.885* | µl/min |
| A_md-renin_ | Scaling factor for macula densa renin secretion | 0.9 | - |
| Aldo_`_ | Aldosterone concentration setpoint | 85 | pg/ml |
| AT1-bound-AngII_0_ | AT1-bound AngII setpoint | 16.6* | pg/ml |
| C_NA,MD,0_ | Macula Densa sodium concentration setpoint | 63.3* | mEq/L |
| G_CO-tpr_ | Proportional gain for cardiac output – TPR controller | 0.1 | - |
| G_Na-vp_ | Proportional gain for vasopressin control of sodium concentration | 0.1 | - |
| K_i-tpr_ | Integral gain for vasopressin control of sodium concentration | 0.1 | - |
| K_i-vp_ | Integral gain for vasopressin control of sodium concentration | 0.005 | - |
| m_aldo,cnt-cd_ | Slope factor for aldosterone effect on CNT/CD sodium reabsorption | 0.5 | - |
| m_aldo,dct_ | Slope factor for aldosterone effect on CNT/CD sodium reabsorption | 0.5 | - |
| m_AT1-aff_ | Slope factor for AT1-bound AngII effect on afferent resistance | 16 | - |
| m_AT1-eff_ | Slope factor for AT1-bound AngII effect on efferent resistance | 16 | - |
| m_AT1-preaff_ | Slope factor for AT1-bound AngII effect on preafferent resistance | 16 | - |
| m_AT1-pt_ | Slope factor for AT1-bound AngII effect on proximal tubule Na+ reabsorption | 16 | - |
| m_autoreg_ | Myogenic autoregulation slope factor | 2 | - |
| m_TGF_ | Tubuloglomerular feedback signal slope factor | 6 | - |
| P_preafferent,0_ | Preafferent arteriole pressure setpoint | 71* | mmHg |
| RIHP_0_ | Renal interstitial hydrostatic pressure setpoint | 9.66* | mmHg |
| S_aldo-cnt-cd_ | Scaling factor for aldosterone effect on CNT/CD sodium reabsorption | 0.2 | - |
| S_aldo-dct_ | Scaling factor for aldosterone effect on DCT sodium reabsorption | 0.05 | - |
| S_AT1-aldo_ | Scaling factor for AT1-bound AngII effect on aldosterone secretion | 0.02 | - |
| S_AT1-aff_ | Scaling factor for AT1-bound AngII effect on afferent resistance | 0.8 | - |
| S_AT1-eff_ | Scaling factor for AT1-bound AngII effect on efferent resistance | 0.8 | - |
| S_AT1-preaff_ | Scaling factor for AT1-bound AngII effect on preafferent resistance | 0.8 | - |
| S_AT1-pt_ | Scaling factor for AT1-bound AngII effect on proximal tubule Na+ reabsorption | 0.1 | - |
| S_autoreg_ | Preafferent autoregulation signal scaling factor | 1 | - |
| S_TGF_ | Tubuloglomerular feedback signal scaling factor | 0.7 | - |
| S_P-N,CNT-DC_ | CNT-DC pressure-natriuresis signal scaling factor | 0.5 | - |
| S_P-N,DCT_ | DCT pressure-natriuresis signal scaling factor | 0.1 | - |
| S_P-N,LoH_ | LoH pressure-natriuresis signal scaling factor | 0.1 | - |
| S_P-N,PT_ | PT pressure-natriuresis signal scaling factor | 0.5 | - |
