# Supplementary material for: Cardiac and renal function interactions in heart failure with reduced ejection fraction: A mathematical modeling analysis
Source: PLoS Comput Biol. 2020 Aug 17;16(8):e1008074. doi: 10.1371/journal.pcbi.1008074 (PMC7451992; doi:10.1371/journal.pcbi.1008074)
Supplement: S5 Table — (DOCX) [file pcbi.1008074.s009.docx]

| **Parameter** | **Definition** | **Value** | **Units** |
| --- | --- | --- | --- |
| ACE | ACE activity | 47.65* | /min |
| Chymase | Chymase activity | 2.5* | /min |
| K_AT1_ | AT1-receptor binding rate | 12.1* | /min |
| K_AT2_ | AT2-receptor binding rate | 4* | /min |
| K_d,AngI_ | AngI degradation rate | 0.0924 | /min |
| K_d,AngI_ | AngII degradation rate | 0.146 | /min |
| K_d,AT1_ | AT1-bound AngII degradation rate | 3.47 | /min |
| K_d,renin_ | Renin degradation rate | 4 | /min |
