# Supplementary material for: Cardiac and renal function interactions in heart failure with reduced ejection fraction: A mathematical modeling analysis
Source: PLoS Comput Biol. 2020 Aug 17;16(8):e1008074. doi: 10.1371/journal.pcbi.1008074 (PMC7451992; doi:10.1371/journal.pcbi.1008074)
Supplement: S1 Appendix — (DOCX) [file pcbi.1008074.s011.docx]

Appendix 1:

The derivation of Equation S69:

The heart was assumed to be spherical, and the myocyte was assumed to be cylindrical.

$A_{o}=4\pi{r_{o}}^{2}=\frac{na^{2}}{4}$ , $r_{o}=\sqrt{\frac{na^{2}}{16\pi}}$

$$V_{o}=\frac{4}{3}\pi{r_{o}}^{3}=\frac{4}{3}\pi{(\frac{a}{4}\sqrt{\frac{n}{\pi}})}^{3}$$

$$V_{new}=\frac{4}{3}\pi{(\frac{\left( a+\Delta a \right)}{4}\sqrt{\frac{n}{\pi}})}^{3}=\frac{4}{3}\pi{(\frac{a\left( 1+\frac{\Delta a}{a} \right)}{4}\sqrt{\frac{n}{\pi}})}^{3}=\frac{4}{3}\pi\left( \frac{a}{4}\sqrt{\frac{n}{\pi}} \right)^{3}\left( 1+\frac{\Delta a}{a} \right)^{3}$$

where r_o_ is the radius of LV cavity at original; A_o_ is the surface area of LV cavity; V_o_ is the LV cavity volume; V_new_ is the LV cavity volume with the change in myocyte length; a is the myocyte length, the myocyte diameter is assumed to be 0.25a. n is the number of myocyte around the cavity. ∆a is the change in myocyte length.
